# Supplementary material for: Whole-genome sequencing reveals genomic signatures associated with the inflammatory microenvironments in Chinese NSCLC patients
Source: Nat Commun. 2018 May 24;9:2054. doi: 10.1038/s41467-018-04492-2 (PMC5967326; doi:10.1038/s41467-018-04492-2)
Supplement: Supplementary file 1 — Supplementary Information [file 41467_2018_4492_MOESM1_ESM.pdf]

Supplementary information

Whole-genome sequencing reveals genomic signatures associated with the inflammatory microenvironments in Chinese NSCLC patients

Wang et al.

Supplementary Figures

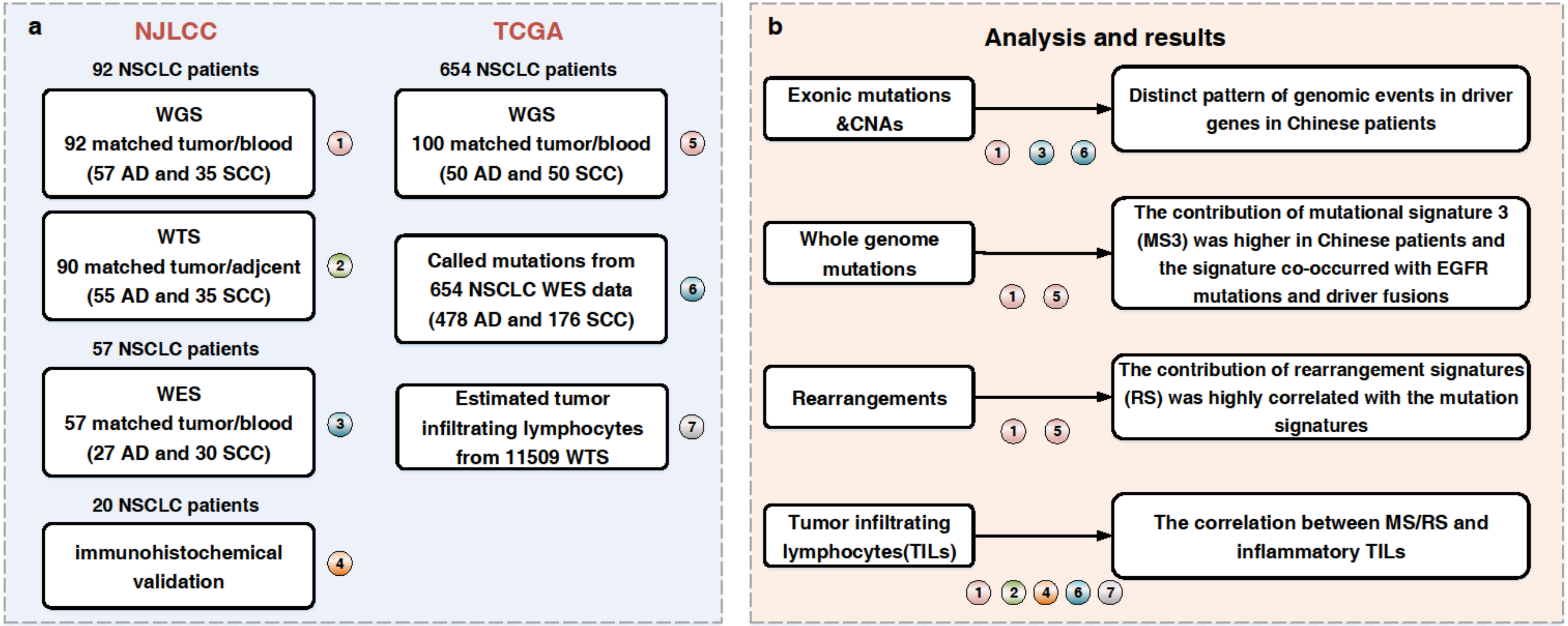

Supplementary Figure 1. General diagram of data and workflow for this study. Whole-genome sequencing of tumor-blood pairs from 92 cases (57 lung AD and 35 lung SCC) was performed. The transcriptome sequences were analyzed in 90 of 92 patients. Additional whole-exome sequencing on 57 pairs (27 lung AD and 30 lung SCC) was used to study exonic mutations. a. The data involved in this study; b. The analysis and results in this study.

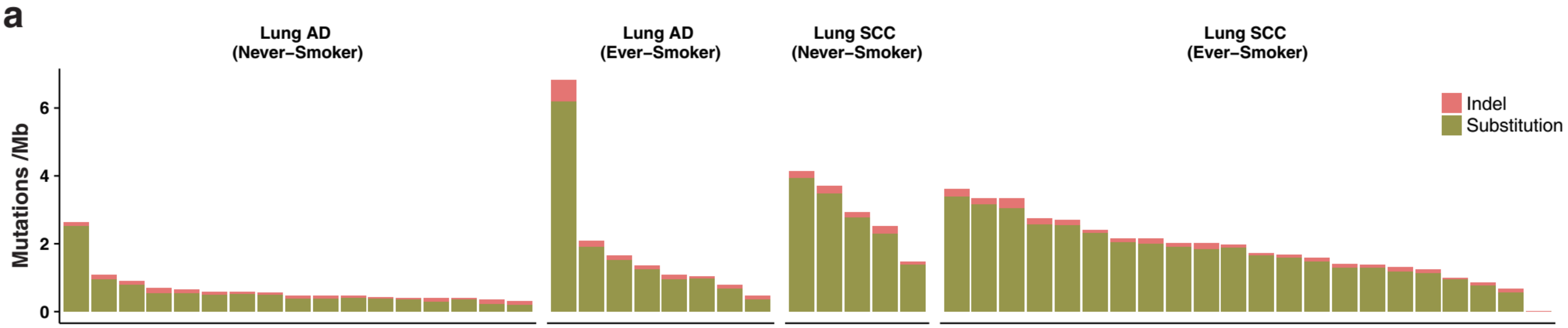
a
Lung AD
(Never–Smoker)
Lung AD
(Ever–Smoker)
Lung SCC
(Never–Smoker)
Lung SCC
(Ever–Smoker)
Mutations /Mb
6
4
2
0
Indel
Substitution

b

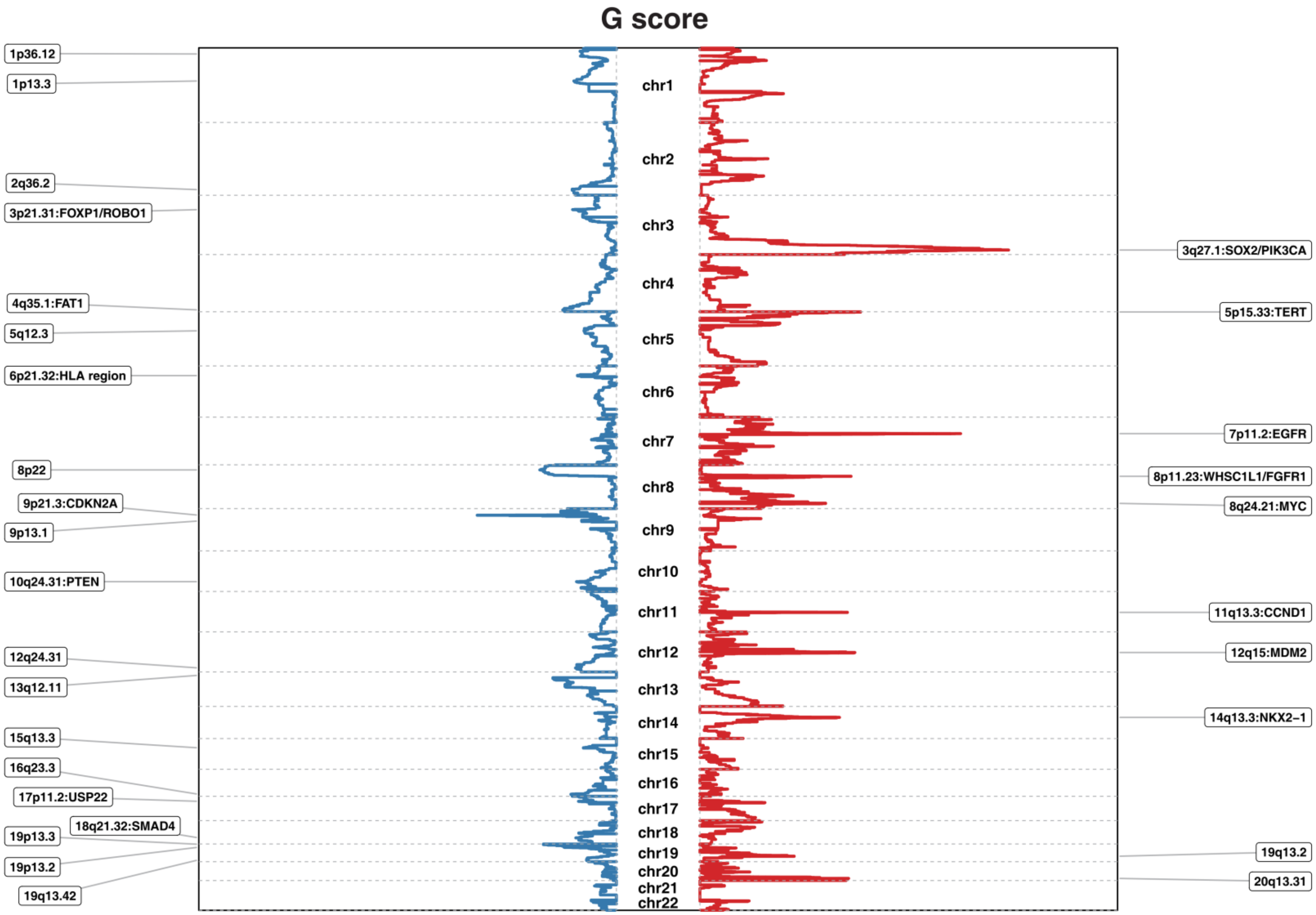

G score
1p36.12
1p13.3
2q36.2
3p21.31:FOXP1/ROBO1
4q35.1:FAT1
5q12.3
6p21.32:HLA region
8p22
9p21.3:CDKN2A
9p13.1
10q24.31:PTEN
12q24.31
13q12.11
15q13.3
16q23.3
17p11.2:USP22
18q21.32:SMAD4
19p13.3
19p13.2
19q13.42
chr1
chr2
chr3
chr4
chr5
chr6
chr7
chr8
chr9
chr10
chr11
chr12
chr13
chr14
chr15
chr16
chr17
chr18
chr19
chr20
chr21
chr22
3q27.1:SOX2/PIK3CA
5p15.33:TERT
7p11.2:EGFR
8p11.23:WHSC1L1/FGFR1
8q24.21:MYC
11q13.3:CCND1
12q15:MDM2
14q13.3:NKX2−1
19q13.2
20q13.31

Supplementary Figure 2. a. Mutation (substitutions and indels) rates identified in the whole-exome sequences data from Chinese NSCLC patients; b. Whole genome distribution of copy number alterations identified using whole-genome sequences data. Red indicates amplifications, and blue indicates deletions. The annotated genes were potential driver genes located in the regions with significantly altered copy numbers detected by GISTIC2.

a

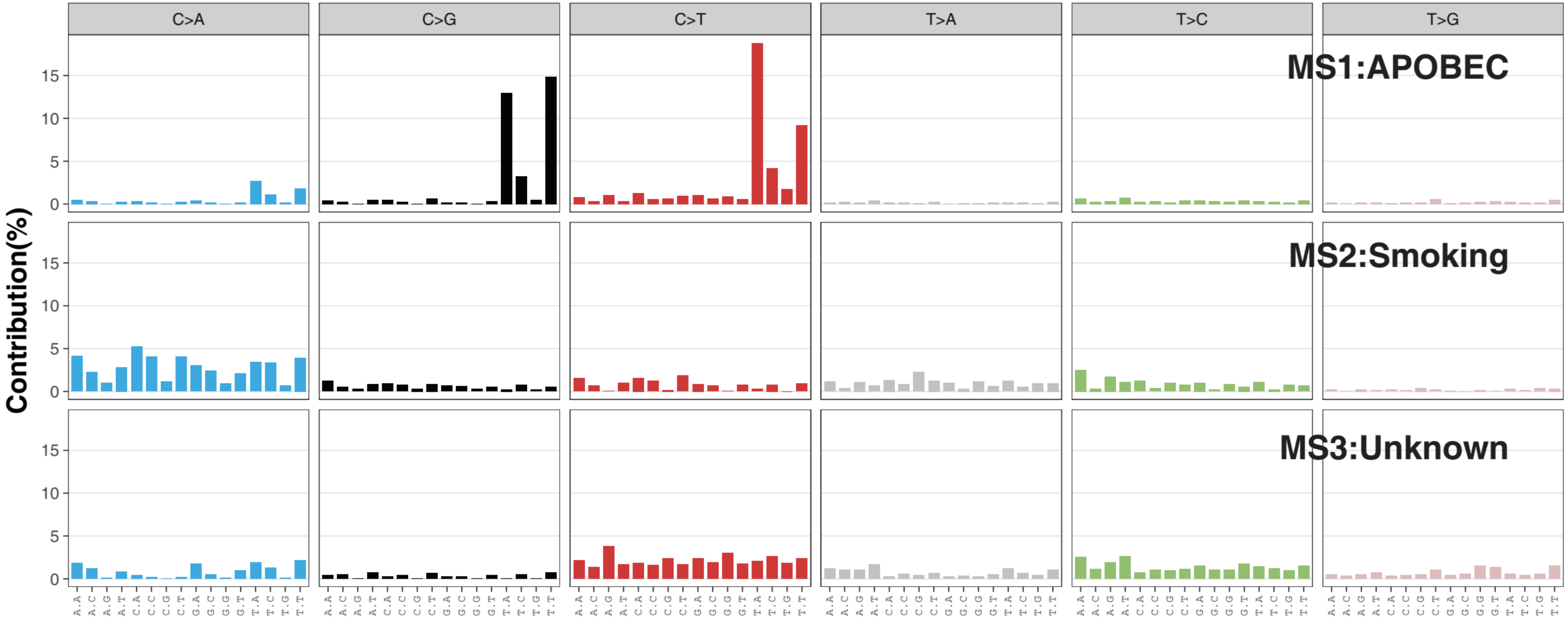
C>A
C>G
C>T
T>A
T>C
T>G
MS1:APOBEC
MS2:Smoking
MS3:Unknown
Contribution(%)
0
5
10
15

b

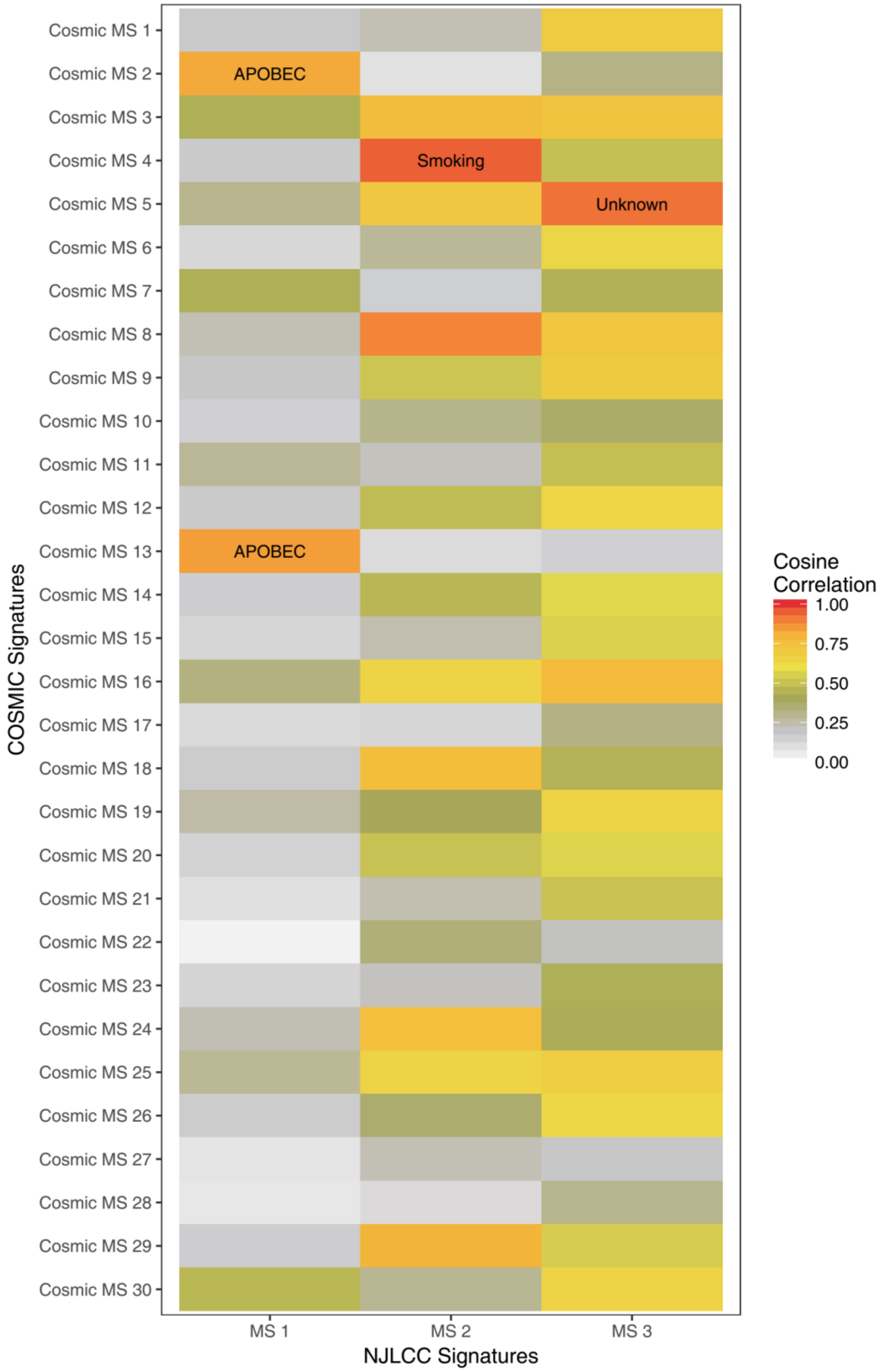
Cosmic MS 1
Cosmic MS 2
Cosmic MS 3
Cosmic MS 4
Cosmic MS 5
Cosmic MS 6
Cosmic MS 7
Cosmic MS 8
Cosmic MS 9
Cosmic MS 10
Cosmic MS 11
Cosmic MS 12
Cosmic MS 13
Cosmic MS 14
Cosmic MS 15
Cosmic MS 16
Cosmic MS 17
Cosmic MS 18
Cosmic MS 19
Cosmic MS 20
Cosmic MS 21
Cosmic MS 22
Cosmic MS 23
Cosmic MS 24
Cosmic MS 25
Cosmic MS 26
Cosmic MS 27
Cosmic MS 28
Cosmic MS 29
Cosmic MS 30
COSMIC Signatures
APOBEC
Smoking
Unknown
APOBEC
MS 1
MS 2
MS 3
NJLCC Signatures
Cosine Correlation
1.00
0.75
0.50
0.25
0.00

c

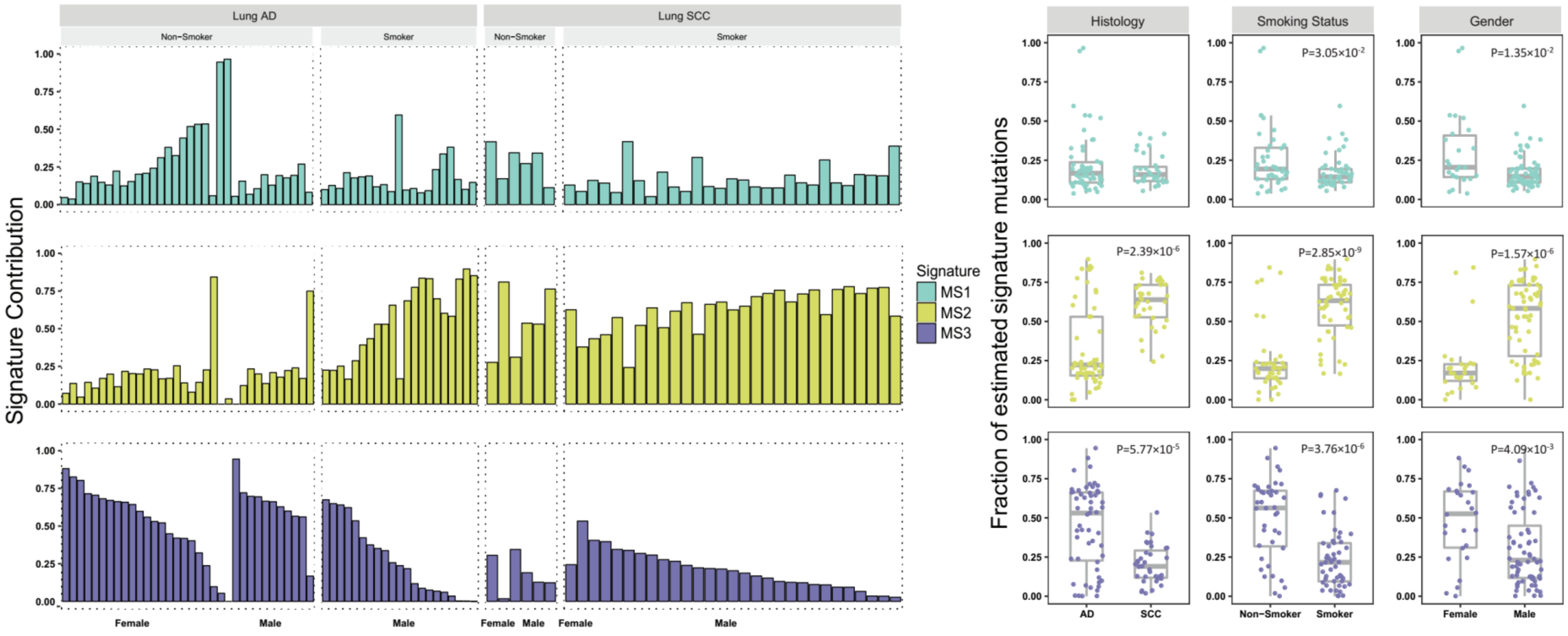

Supplementary Figure 3. a. Three mutational signatures identified in our patients; b. Correlation between the mutational signatures derived from the NJLCC patients and previously defined signatures from COSMIC. A pair-wise cosine correlation was performed between the COSMIC and NJLCC signatures. The top one or two most correlated COSMIC signatures were used to determine the identity of each NJLCC signature, and

each NJLCC signature was renamed accordingly. c. Contribution of each NJLCC signature in all samples and their associations with histology, smoking status and gender. The Wilcoxon rank sum test was used to evaluate the differences.

a

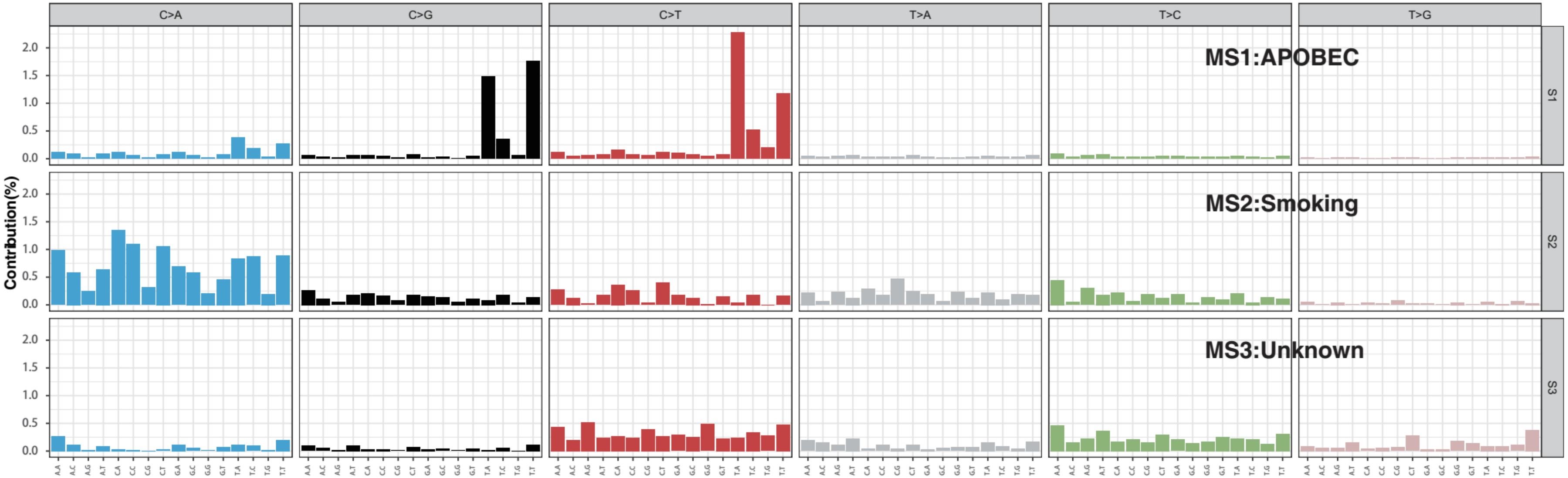
C>A
C>G
C>T
T>A
T>C
T>G
MS1:APOBEC
MS2:Smoking
MS3:Unknown
S1
S2
S3
Contribution(%)
2.0
1.5
1.0
0.5
0.0

b

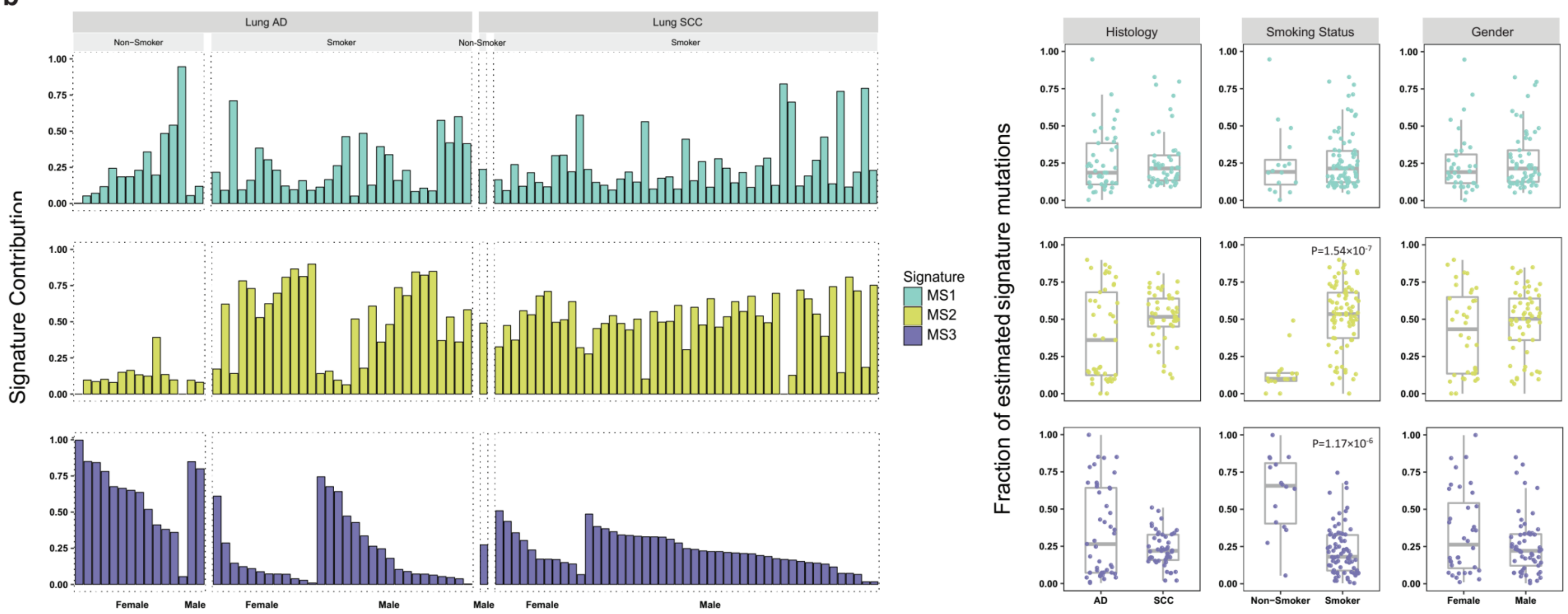

Supplementary Figure 4. a. Three similar mutational signatures extracted from whole-genome sequencing data from 100 TCGA NSCLC patients. b. Contribution of each TCGA NSCLC signature in all samples and their associations with histology, smoking status and gender. The Wilcoxon rank sum test was used to evaluate the differences.

a

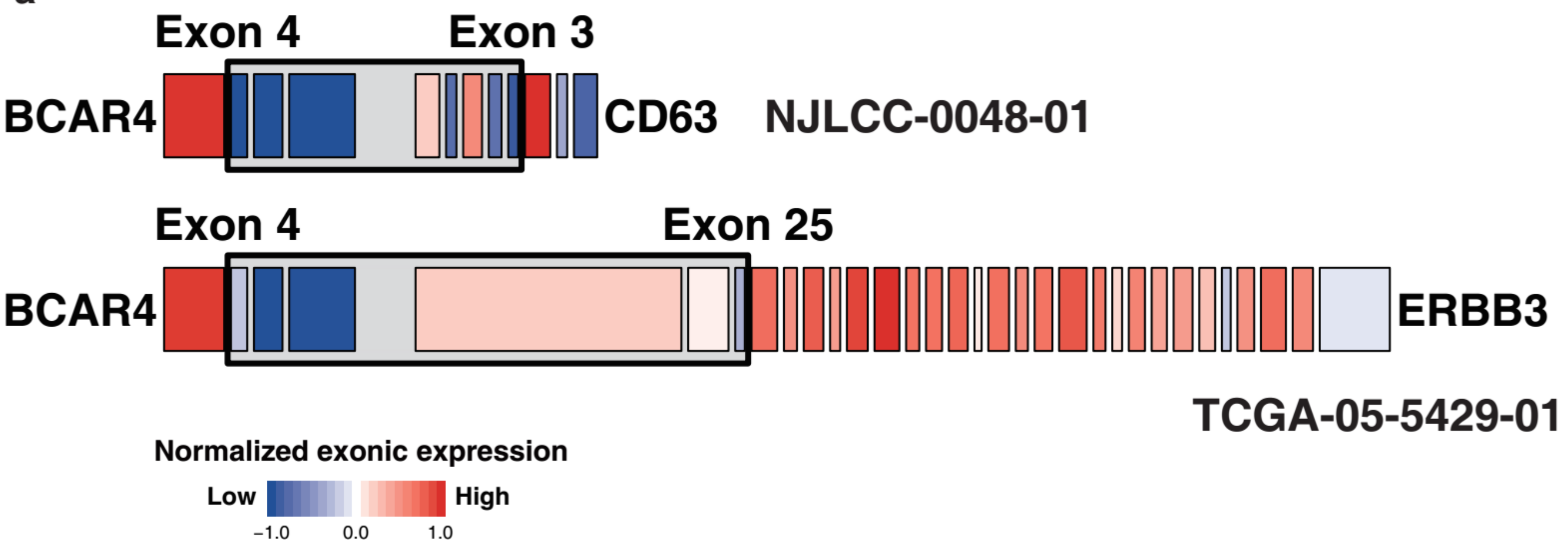
Exon 4
Exon 3
BCAR4
CD63
NJLCC-0048-01
Exon 4
Exon 25
BCAR4
ERBB3
TCGA-05-5429-01
Normalized exonic expression
Low
High
−1.0
0.0
1.0

b

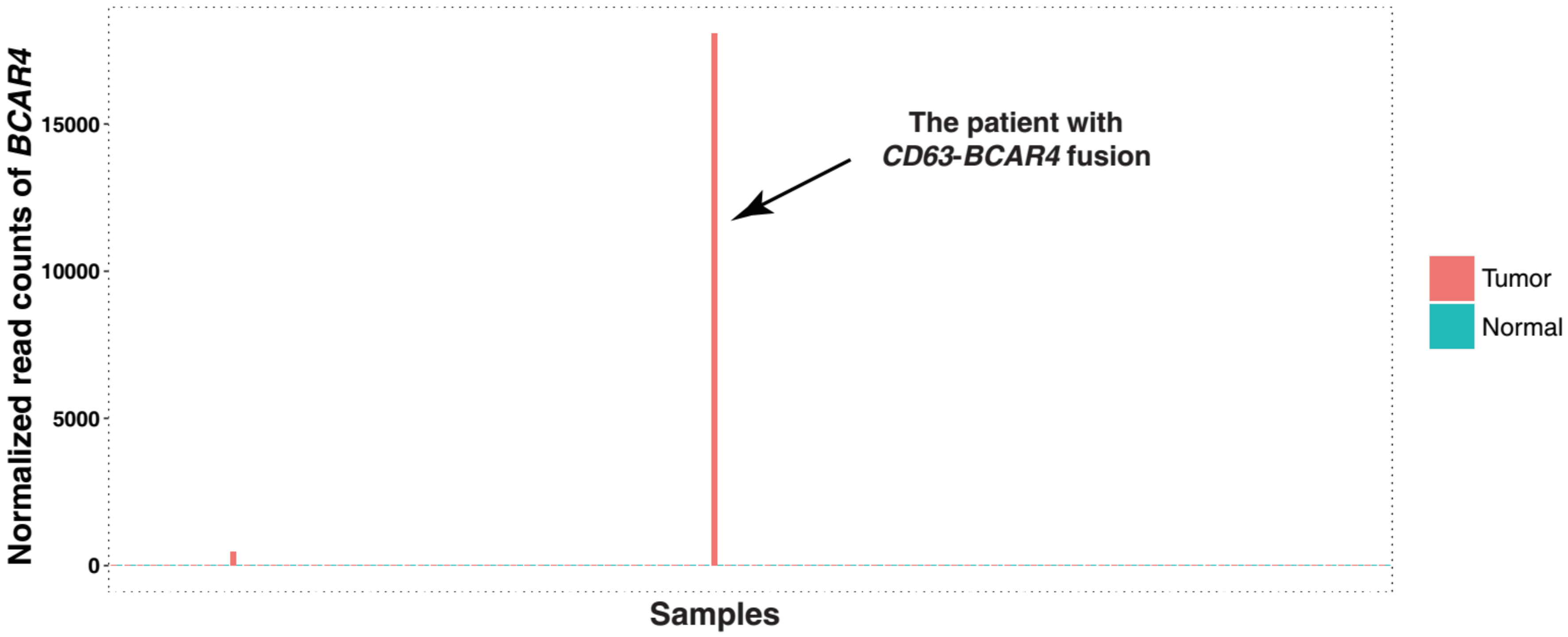
Normalized read counts of BCAR4
15000
10000
5000
0
The patient with
CD63-BCAR4 fusion
Tumor
Normal
Samples

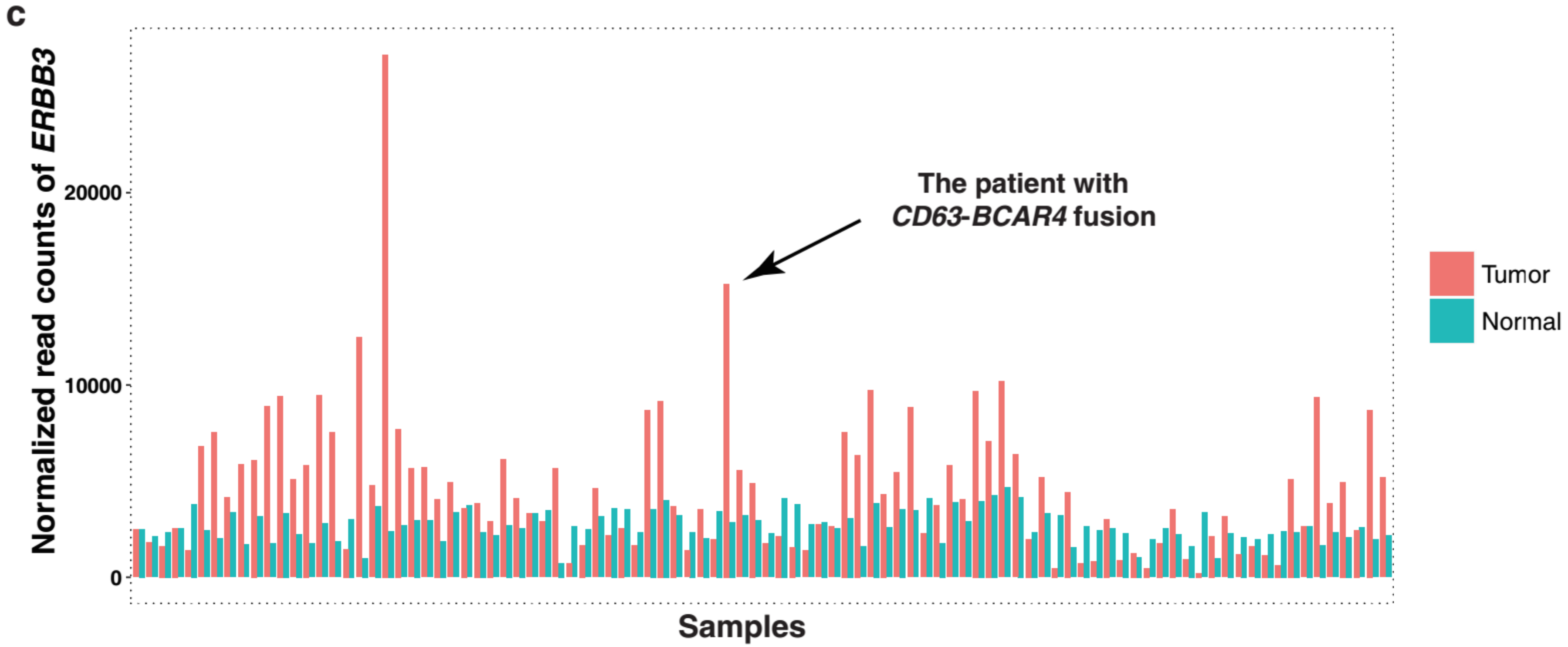

Supplementary Figure 5. a. The fusions involving *BCAR4* occurred in one our patient and one TCGA patient. The expression of exons retained in the putative fusion transcript was relatively higher than the expression of exons not in the putative fusion transcript (as indicated by the gray box). b. The expression of *BCAR4* was highly activated in our patient. c. The expression of *ERBB3* was highly activated in our patient.

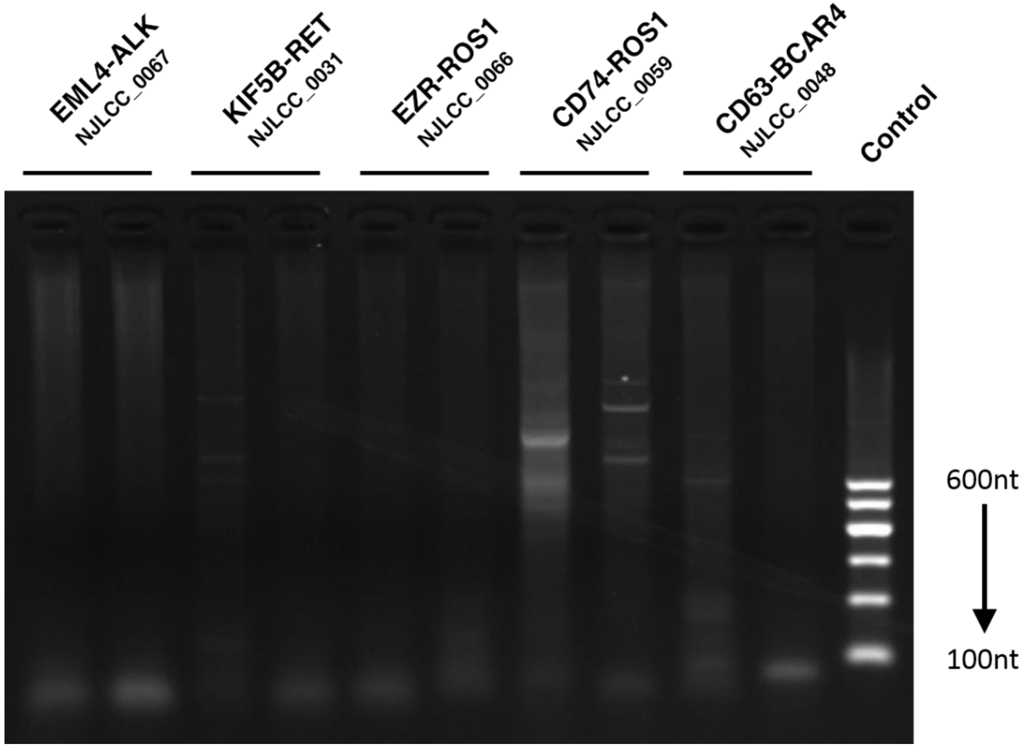

Supplementary Figure 6. Electrophoretogram of rearrangements PCR products in adjacent normal tissues

a

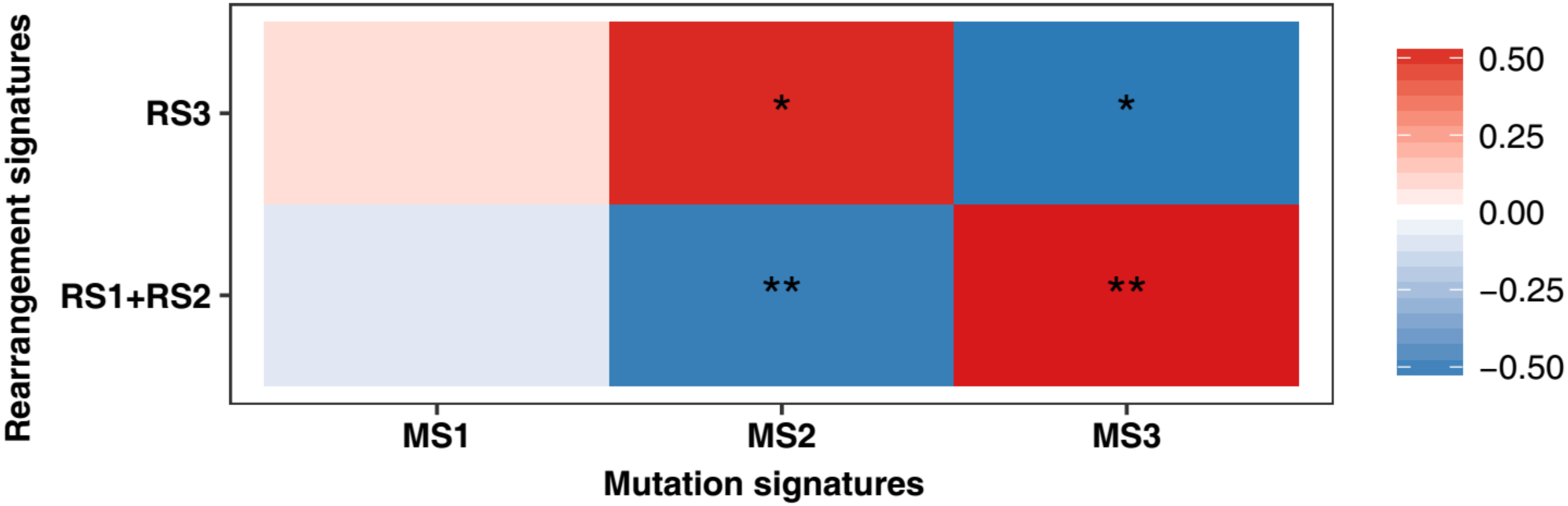
Rearrangement signatures
RS3
RS1+RS2
*
*
**
**
MS1
MS2
MS3
Mutation signatures
0.50
0.25
0.00
−0.25
−0.50

b

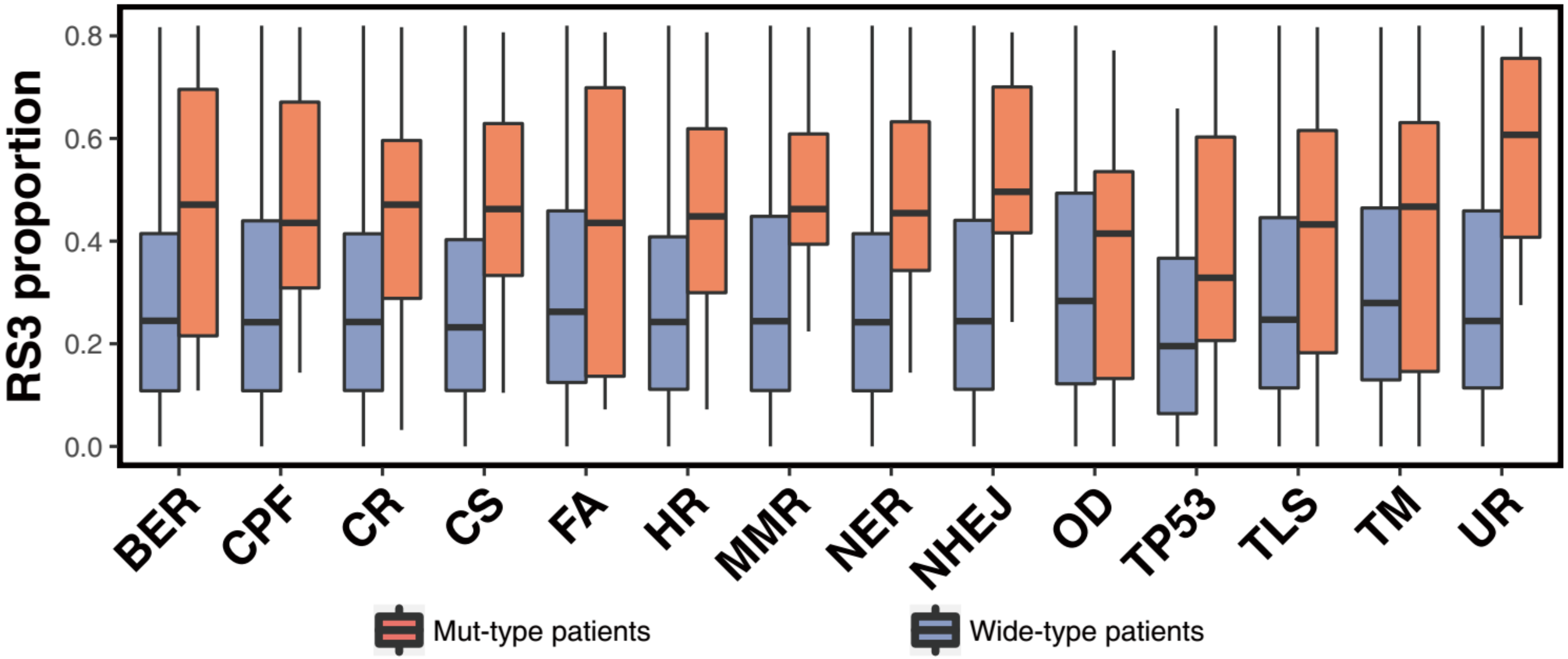
RS3 proportion
0.0
0.2
0.4
0.6
0.8
BER
CPF
CR
CS
FA
HR
MMR
NER
NHEJ
OD
TP53
TLS
TM
UR
Mut-type patients
Wide-type patients

c

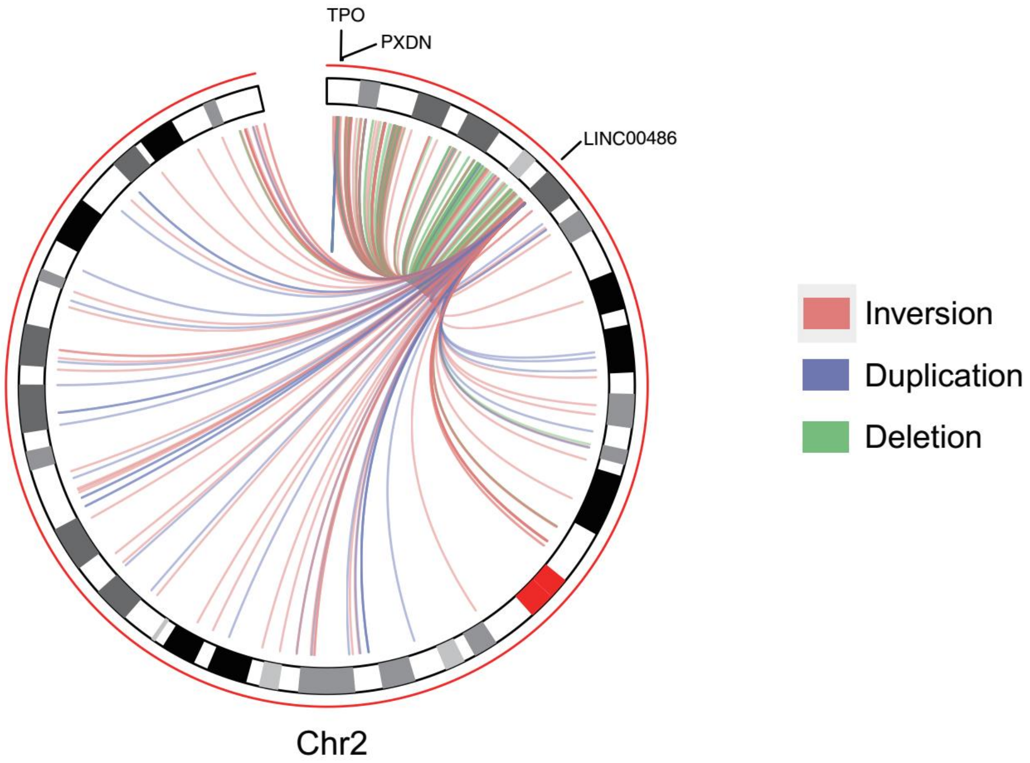

Supplementary Figure 7. a. Correlation matrix between the NJLCC rearrangement signatures and the NJLCC mutational signatures. ***: $P<0.001$; **: $P<0.01$; *: $P<0.05$; b. Association between mutations in the sub-pathways and the RS3 rearrangement proportion (AM, alternative mechanism for telomere maintenance; BER, base excision repair; CPF, checkpoint factor; CR, chromatin remodeling; CS, chromosome segregation; FA, Fanconi anemia pathway; HR, homologous recombination; MMR, mismatch repair; NER, nucleotide excision repair; NHEJ, nonhomologous end joining; OD, other double-strand break repair; TLS, translesion synthesis; TM, telomere maintenance; UR, ubiquitylation response). c. Two new potential fragile sites with hyper-rearrangements in NSCLC patients.

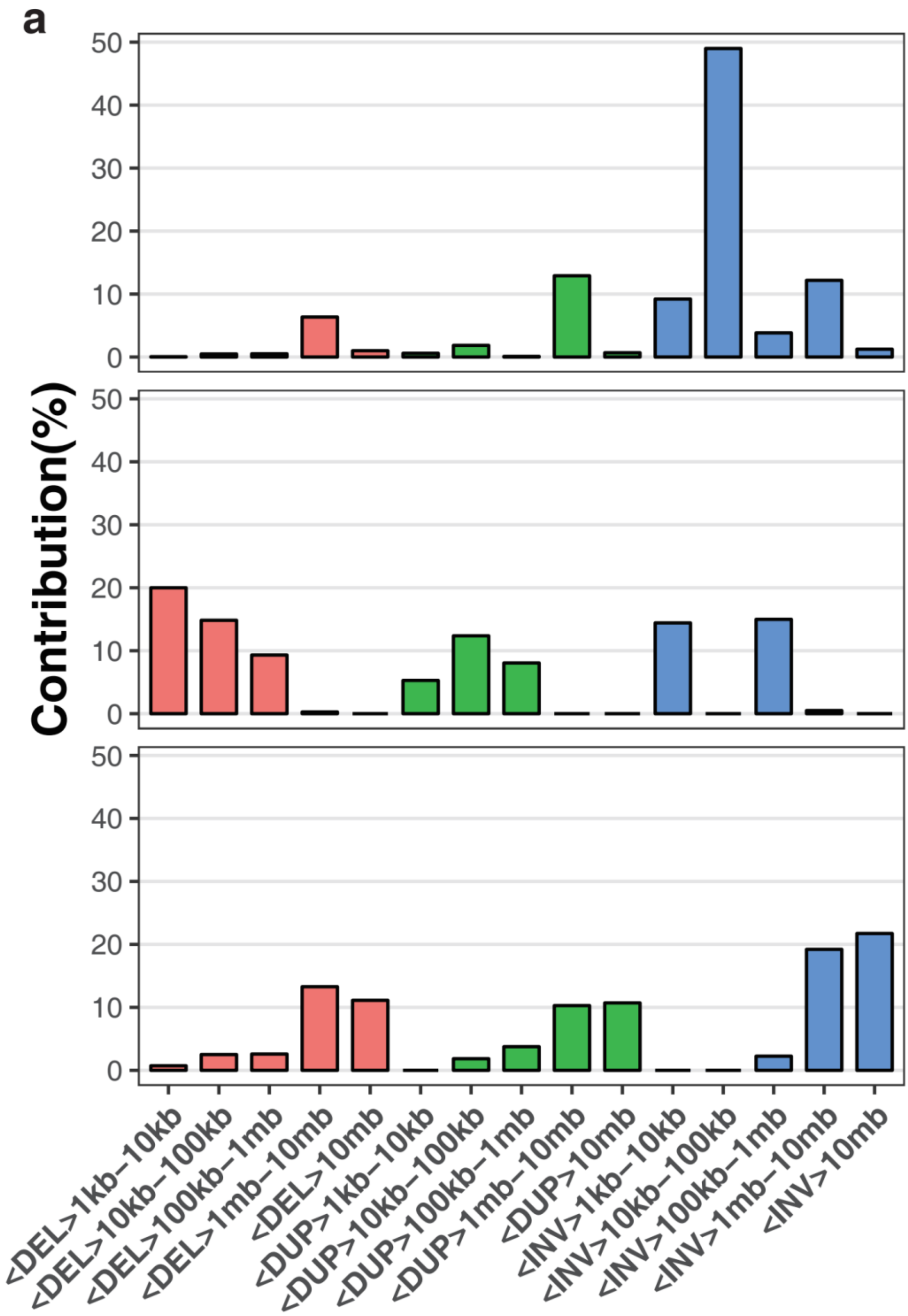
a
Contribution(%)
0
10
20
30
40
50
<DEL>1kb–10kb
<DEL>10kb–100kb
<DEL>100kb–1mb
<DEL>1mb–10mb
<DEL>10mb
<DUP>1kb–10kb
<DUP>10kb–100kb
<DUP>100kb–1mb
<DUP>1mb–10mb
<DUP>10mb
<INV>1kb–10kb
<INV>10kb–100kb
<INV>100kb–1mb
<INV>1mb–10mb
<INV>10mb

b

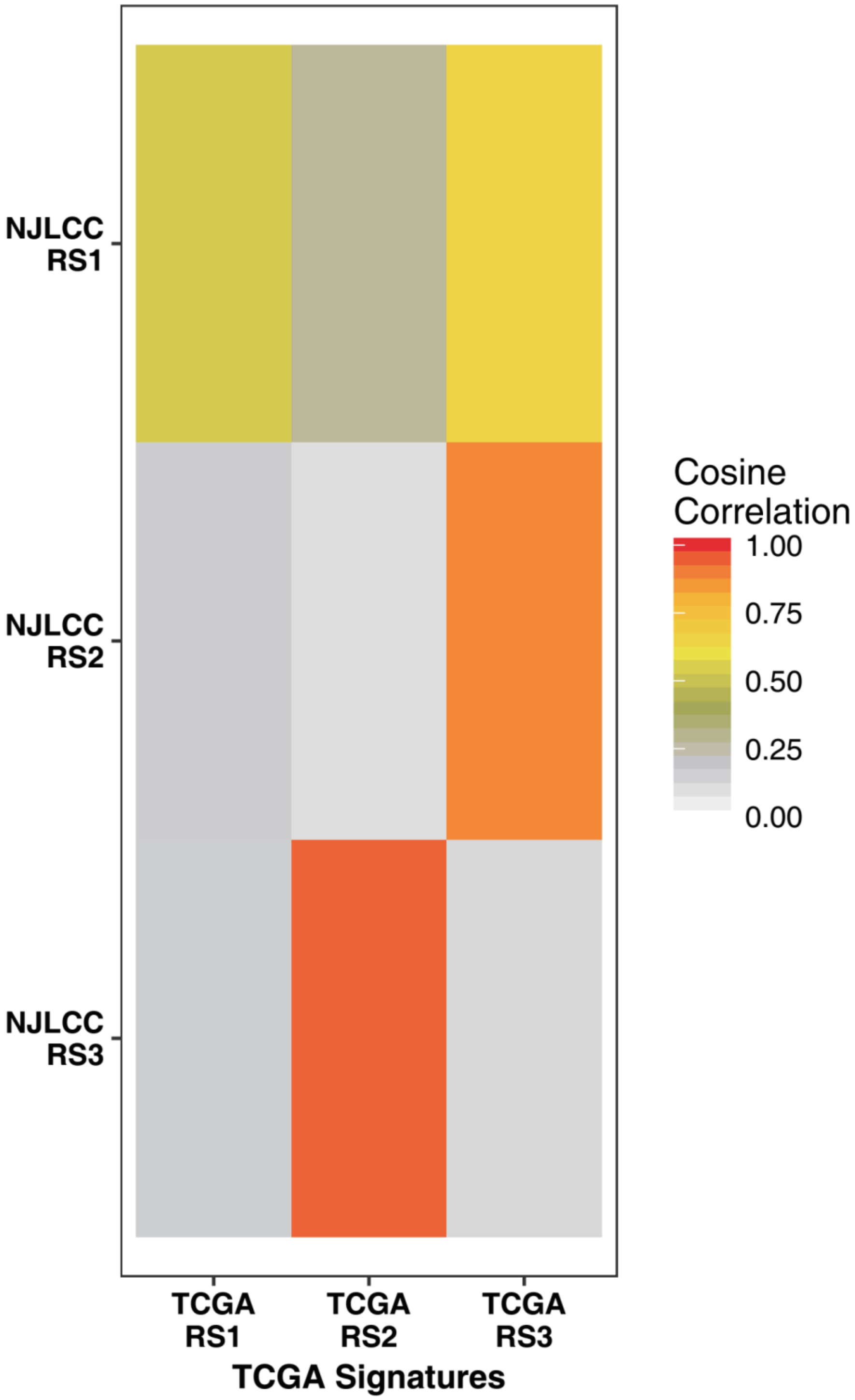

Supplementary Figure 8. a. Three rearrangement signatures extracted from whole-genome sequencing data from 100 TCGA NSCLC patients. b. Correlation between NJLCC rearrangement signatures and TCGA rearrangement signatures.

a

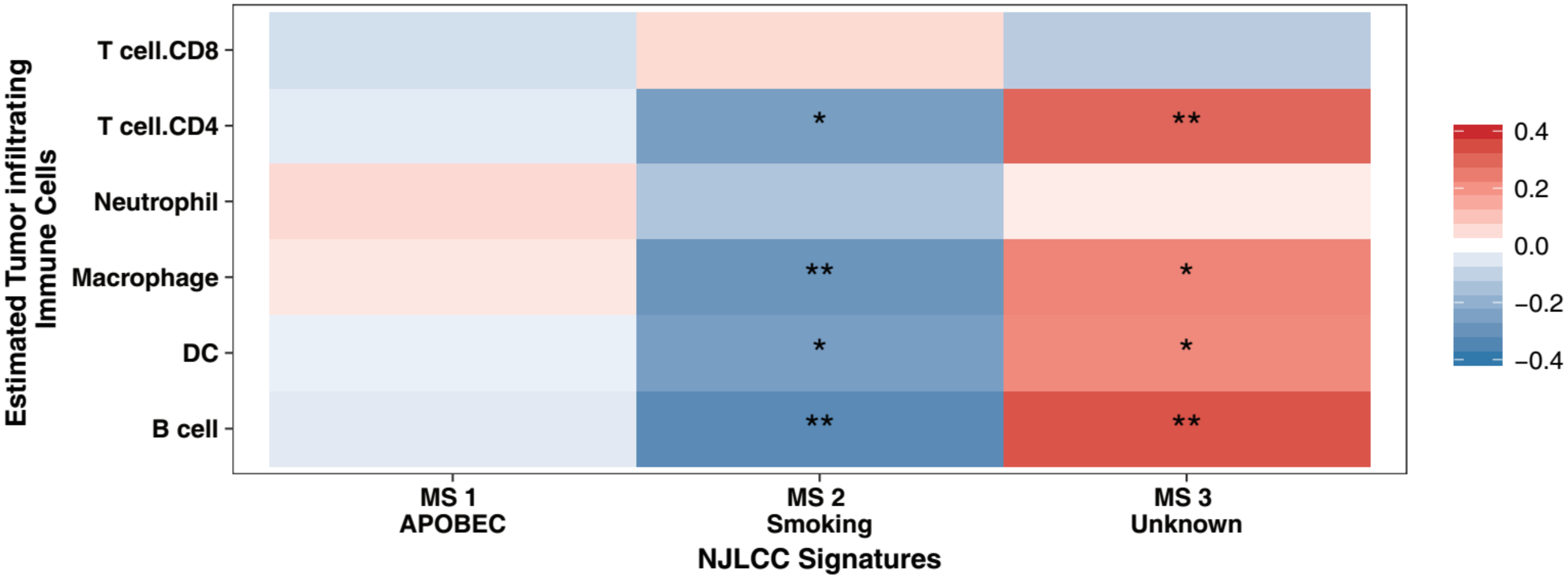
Estimated Tumor infiltrating Immune Cells
T cell.CD8
T cell.CD4
Neutrophil
Macrophage
DC
B cell
*
**
**
*
*
*
**
**
0.4
0.2
0.0
−0.2
−0.4
MS 1 APOBEC
MS 2 Smoking
MS 3 Unknown
NJLCC Signatures

b

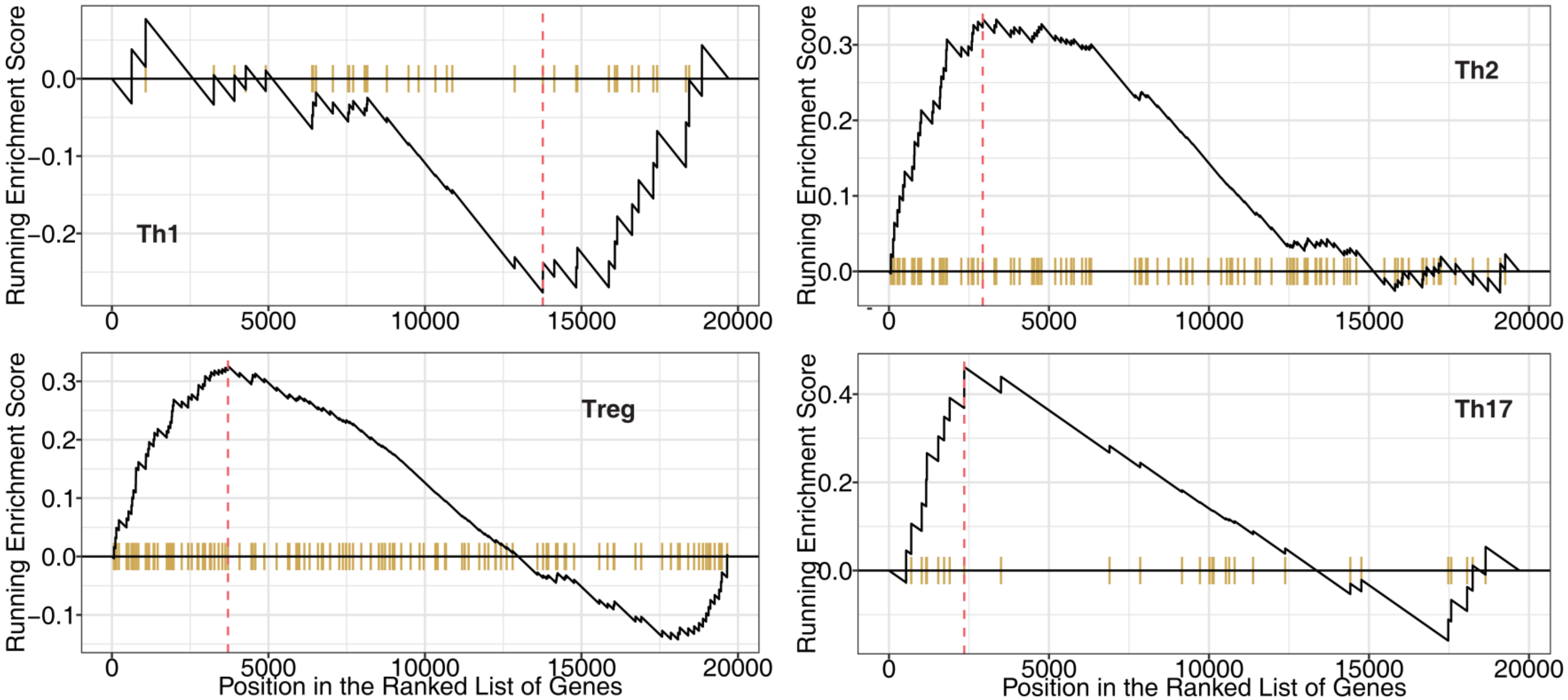

Running Enrichment Score
Th1
Th2
Treg
Th17
Position in the Ranked List of Genes

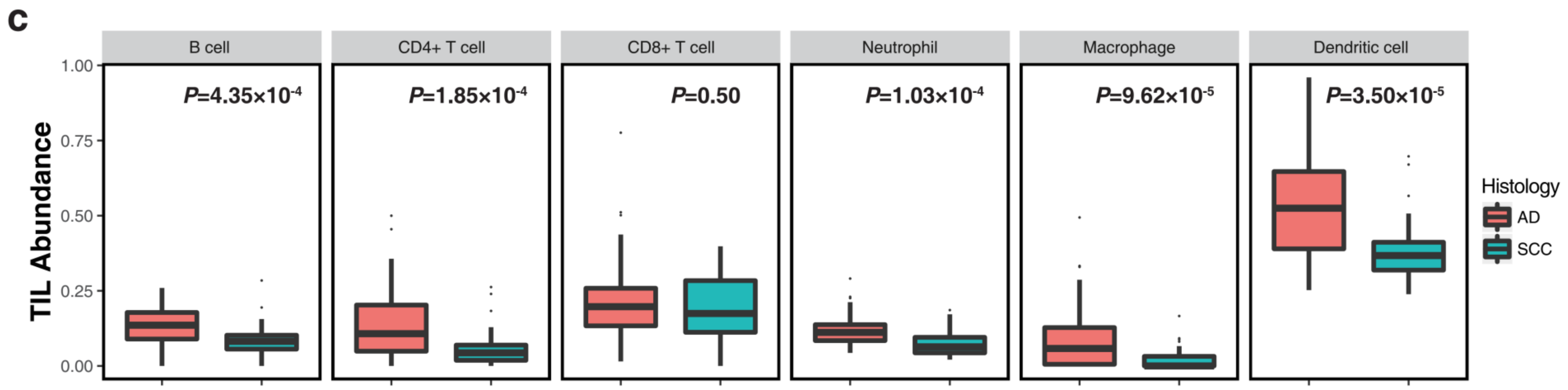
c
B cell
CD4+ T cell
CD8+ T cell
Neutrophil
Macrophage
Dendritic cell
P=4.35×10-4
P=1.85×10-4
P=0.50
P=1.03×10-4
P=9.62×10-5
P=3.50×10-5
TIL Abundance
1.00
0.75
0.50
0.25
0.00
Histology
AD
SCC

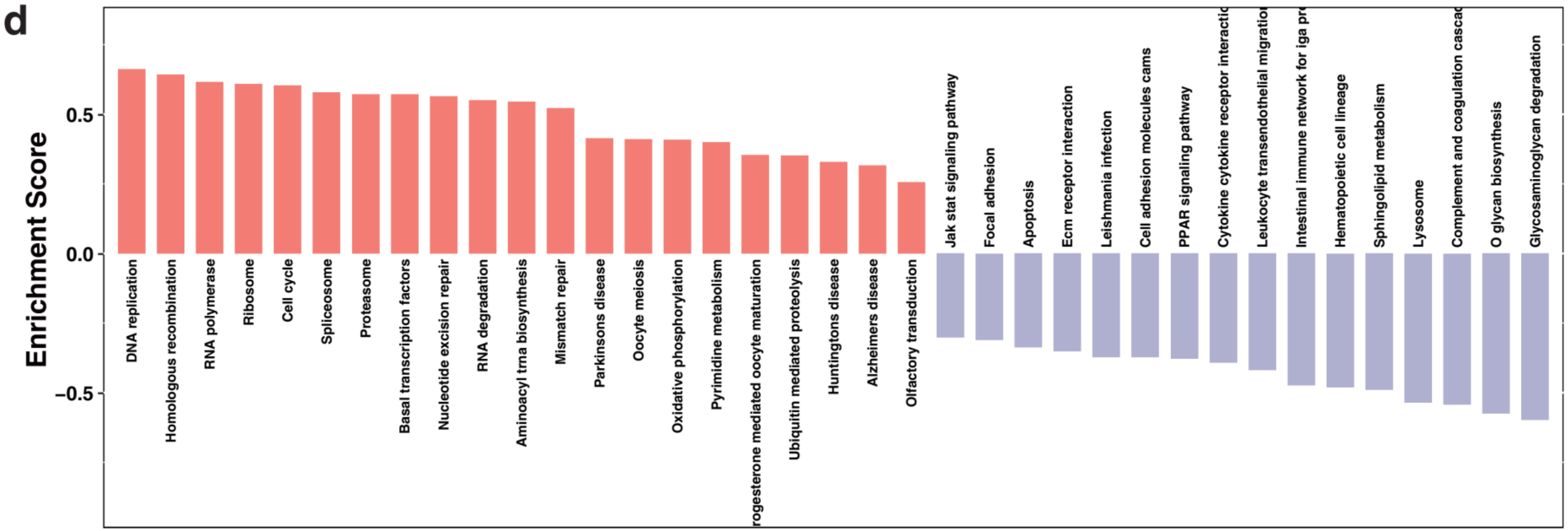

Supplementary Figure 9. a. Correlation matrix between NJLCC mutational signatures and the abundance of TILs. ***: P<0.001; **: P<0.01; *: P<0.05; b. The distribution of enrichment scores in GSEA analysis with Th1, Th2, Th17 and Treg cell-specific gene sets; c. Association between the TIL abundance and NSCLC histology; d. The GSEA analysis revealed that genes positively correlated with MS2 were enriched in DNA replication and damage response pathways.

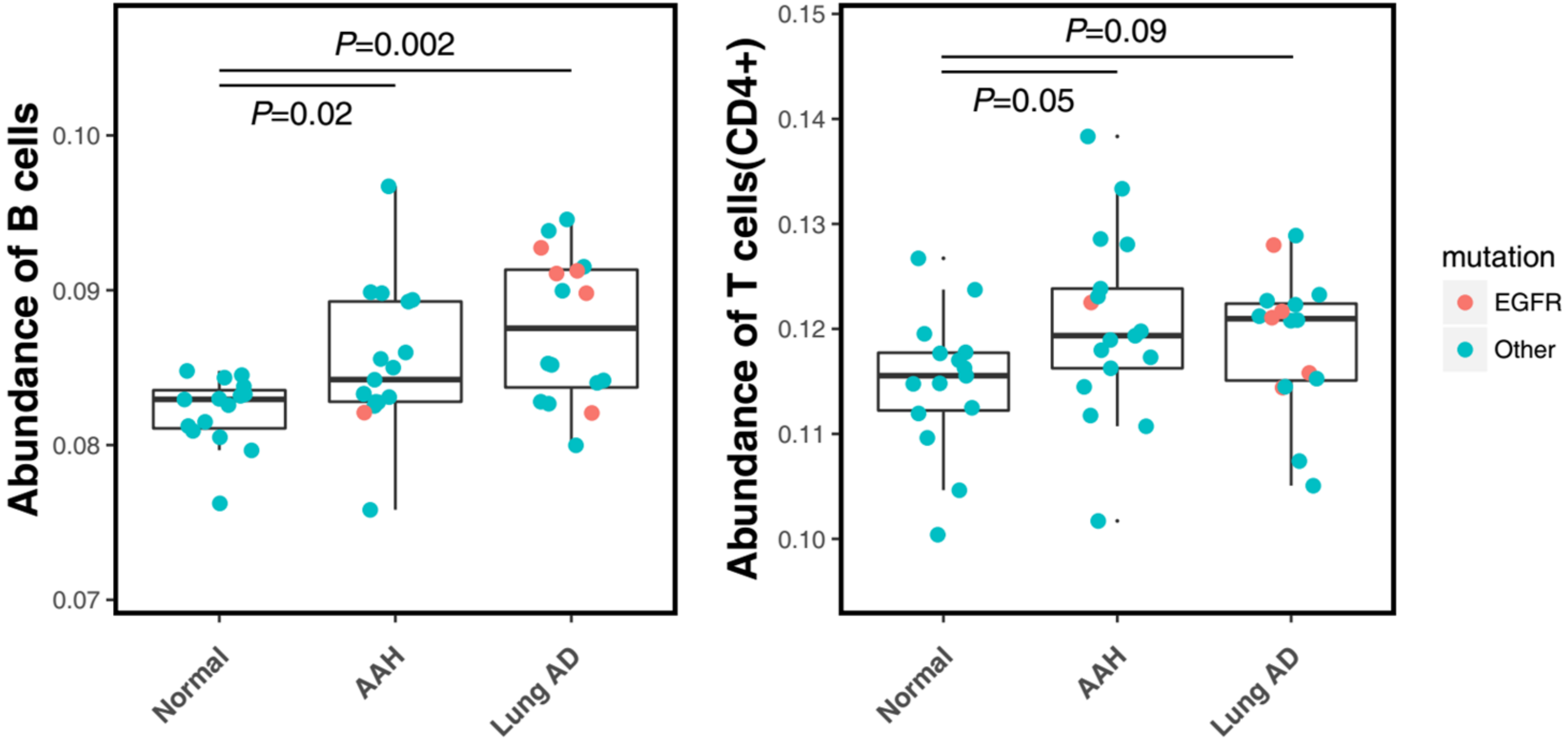

Supplementary Figure 10. Abundance of B cells and T cells (CD4+) in normal, AAH (atypical adenomatous hyperplasia) and Lung AD. Samples with *EGFR* mutations were colored as red.

Supplementary Tables

Supplementary Table 1. General Description of Samples.

	NJLCC WGS		NJLCC WES		TCGA WGS		TCGA WES	
	Lung AD (n=57)	Lung SCC (n=35)	Lung AD (n=27)	Lung SCC (n=30)	Lung AD (n=50)	Lung SCC (n=50)	Lung AD (n=478)	Lung SCC (n=176)
Age (median; range)	60.0 (34.0-77.0)	63.0 (37.0-75.0)	59.0 (32.5-75.3)	60.9 (46.9-75.0)	66.0 (41.0-82.0)	68.0 (47.0-83.0)	67.0 (38.0-88.0)	68.0 (40.0-85.0)
Gender								
Male	32 (56.1)	32 (91.4)	13 (48.1)	30 (100)	24 (48.0)	38 (76.0)	221 (46.2)	130 (73.9)
Female	25 (43.9)	3 (8.6)	14 (51.9)	0 (0)	26 (52.0)	12 (24.0)	257 (53.8)	46 (26.1)
N/A	n/a	n/a	n/a	n/a	n/a	n/a	n/a	n/a
Smoking History								
Ever smoker	21 (36.8)	29 (82.9)	8 (29.6)	22 (73.3)	31 (62.0)	48 (96.0)	394 (82.4)	165(93.8)
Lifelong non-smoker	36 (63.2)	6 (17.1)	17 (63.0)	5 (16.7)	16 (32.0)	1 (2.0)	70 (14.6)	6 (3.4)
N/A	n/a	n/a	2 (7.4)	3 (10.0)	3 (6.0)	1 (2.0)	14 (2.9)	5 (2.8)
Stage								
I	21 (36.8)	12 (34.3)	2 (7.4)	3 (10.0)	21 (42.0)	26 (52.0)	259 (54.2)	97(55.1)
II	16 (28.1)	16 (45.7)	8 (29.6)	14 (46.7)	13 (26.0)	13 (26.0)	113 (23.6)	40 (22.7)
III-IV	20 (35.1)	7 (20.0)	17 (63.0)	13 (43.3)	16 (32.0)	11 (22.0)	104 (21.8)	39 (22.2)
N/A	n/a	n/a	n/a	n/a	n/a	n/a	2(0.4)	n/a

WGS: whole genome sequencing; WES: whole exome sequencing; Lung AD: lung adenocarcinoma; Lung SCC: lung squamous cell carcinoma.

Supplementary Table 2. Significantly mutated protein-coding genes identified in NSCLC.

SYMBOL	SIGNALS*	SIGNAL COUNT	MUT SAMPLES	OncodriveCLUST		OncodriveFM		MutSigCV	
				p-value	q-value	p-value	q-value	p-value	q-value
**EGFR**	CFR	3	48	1.41E-03	**5.63E-03**	0.00E+00	**0.00E+00**	2.05E-08	**9.66E-05**
**PTEN**	CFR	3	7	1.10E-02	**2.93E-02**	2.15E-10	**8.18E-08**	1.43E-05	**2.70E-02**
**TP53**	FR	2	91	5.53E-02	8.85E-02	0.00E+00	**0.00E+00**	0.00E+00	**0.00E+00**
**NFE2L2**	FR	2	18	5.28E-02	8.85E-02	0.00E+00	**0.00E+00**	1.06E-12	**6.68E-09**
**RB1**	FR	2	19			0.00E+00	**0.00E+00**	3.69E-08	**1.39E-04**
**CDKN2A**	FR	2	18			4.33E-15	**2.63E-12**	0.00E+00	**0.00E+00**
**KRAS**	CF	2	7	8.74E-08	**6.99E-07**	3.26E-06	**9.91E-04**	4.65E-04	3.56E-01
**KMT2D**	F	1	23			2.91E-11	**1.48E-08**	1.33E-04	1.40E-01
**STK11**	R	1	8			1.10E-01	5.98E-01	1.02E-05	**2.13E-02**
TET2	F	1	7			4.36E-04	**4.29E-02**	6.46E-01	1.00E+00
**FBXW7**	F	1	6			7.22E-11	**3.14E-08**	2.36E-03	9.89E-01
**ARID1A**	F	1	5			9.71E-08	**3.28E-05**	1.97E-02	1.00E+00
**SETD2**	F	1	5			1.11E-04	**1.69E-02**	8.78E-01	1.00E+00
**PBRM1**	F	1	3			4.51E-04	**4.29E-02**	1.88E-01	1.00E+00
FLNC	F	1	7			1.60E-04	**2.21E-02**	4.82E-01	1.00E+00
**ARHGAP35**	F	1	7			3.67E-05	**8.60E-03**	4.84E-01	1.00E+00
**CLASP2**	F	1	7			1.43E-04	**2.06E-02**	9.98E-01	1.00E+00
WNK1	F	1	5			1.66E-05	**4.21E-03**	9.62E-01	1.00E+00
AGAP1	F	1	5			7.81E-05	**1.41E-02**	3.65E-01	1.00E+00
POLE	F	1	5			3.74E-04	**4.06E-02**	3.36E-02	1.00E+00
OBSL1	F	1	4			5.54E-05	**1.20E-02**	5.41E-01	1.00E+00
TDG	F	1	4			7.90E-05	**1.41E-02**	4.13E-03	1.00E+00
SEMA6B	F	1	4			4.44E-04	**4.29E-02**	2.94E-02	1.00E+00

ITGAL	F	1	3		9.63E-05	**1.54E-02**	3.14E-01	1.00E+00
EEF1D	F	1	3		2.76E-04	**3.22E-02**	6.95E-02	1.00E+00
NT5E	F	1	3		8.45E-06	**2.34E-03**	1.30E-01	1.00E+00
PFKM	F	1	3		2.18E-04	**2.88E-02**	4.37E-01	1.00E+00

*: C, OncodriveCLUST; F, OncodriveFM; R, Recurrent

Bold: potential driver genes

Supplementary Table 3. Significantly mutated protein-coding genes identified in lung adenocarcinoma.

SYMBOL	SIGNALS*	SIGNAL COUNT	MUT SAMPLES	OncodriveCLUST		OncodriveFM		MutSigCV	
				p-value	q-value	p-value	q-value	p-value	q-value
**EGFR**	CFR	3	44	5.12E-04	**7.68E-04**	0.00E+00	**0.00E+00**	3.11E-15	**2.93E-11**
**TP53**	FR	2	41	3.21E-01	3.21E-01	0.00E+00	**0.00E+00**	7.77E-16	**1.47E-11**
**RB1**	FR	2	13			2.03E-13	**6.82E-11**	6.00E-15	**3.77E-11**
**KRAS**	CF	2	7	8.74E-08	**2.62E-07**	5.04E-06	**1.27E-03**	1.38E-04	4.34E-01
**SMARCA4**	F	1	4			1.13E-04	**1.28E-02**	2.81E-01	1.00E+00
**SETD2**	F	1	3			9.29E-05	**1.28E-02**	7.87E-01	1.00E+00
WNK1	F	1	4			9.08E-05	**1.28E-02**	9.90E-01	1.00E+00
EPRS	F	1	4			3.89E-04	**3.93E-02**	9.67E-01	1.00E+00
TDG	F	1	3			1.14E-04	**1.28E-02**	1.42E-03	1.00E+00

*: C, OncodriveCLUST; F, OncodriveFM; R, Recurrent

Bold: potential driver genes

Supplementary Table 4. Significantly mutated protein-coding genes identified in lung squamous cell carcinoma.

SYMBOL	SIGNALS*	SIGNAL COUNT	MUT SAMPLES	OncodriveCLUST		OncodriveFM		MutSigCV	
				p-value	q-value	p-value	q-value	p-value	q-value
**TP53**	CFR	3	51	3.82E-03	**1.15E-02**	0.00E+00	**0.00E+00**	1.11E-16	**2.09E-12**
**NFE2L2**	FR	2	18	5.28E-02	7.92E-02	0.00E+00	**0.00E+00**	7.48E-14	**4.70E-10**
**KMT2D**	FR	2	20			4.06E-12	**2.30E-09**	3.81E-07	**1.20E-03**
**CDKN2A**	FR	2	14			3.32E-11	**9.91E-09**	1.37E-14	**1.29E-10**
**FBXW7**	F	1	6			6.74E-11	**1.64E-08**	3.95E-04	4.38E-01
**RB1**	F	1	5			5.45E-08	**1.16E-05**	2.36E-01	1.00E+00
TET2	F	1	5			2.22E-04	**2.70E-02**	1.28E-01	1.00E+00
**PTEN**	F	1	5			5.53E-12	**2.35E-09**	2.54E-04	3.68E-01
**ARID1A**	F	1	4			1.18E-06	**2.24E-04**	5.65E-02	1.00E+00
**PBRM1**	F	1	2			4.63E-04	**4.15E-02**	3.59E-01	1.00E+00
**FAT1**	F	1	12			3.49E-11	**9.91E-09**	3.84E-01	1.00E+00
**SVEP1**	F	1	6			1.15E-04	**1.50E-02**	9.97E-01	1.00E+00
AGAP1	F	1	4			2.81E-05	**4.78E-03**	3.96E-01	1.00E+00
SEMA6B	F	1	4			4.06E-04	**4.06E-02**	1.73E-02	1.00E+00
ITGAL	F	1	3			7.22E-05	**1.02E-02**	1.44E-01	1.00E+00
EEF1D	F	1	3			2.91E-04	3.31E-02	5.12E-02	**1.00E+00**

*: C, OncodriveCLUST; F, OncodriveFM; R, Recurrent

Bold: potential driver genes

Supplementary Table 5. Copy number alterations identified in NSCLC patients.

Unique Name	Descriptor	Wide Peak Limits	q values	Residual q values	Number of genes in peak	Number of microRNAs in peak	Top candidate genes in wide peak
Amplification Peak 1	3q27.1	chr3:176696138-183772886	1.63E-10	1.63E-10	52	1	SOX2/PIK3CA
Amplification Peak 2	5p15.33	chr5:1-2888978	5.56E-04	5.56E-04	29	4	TERT
Amplification Peak 3	7p11.2	chr7:54651541-55777338	7.74E-09	7.74E-09	6	0	EGFR
Amplification Peak 4	8p11.23	chr8:37416926-41158567	1.43E-03	1.43E-03	37	1	WHSC1L1/FGFR1
Amplification Peak 5	8q24.21	chr8:121797717-131083276	1.32E-02	1.32E-02	52	8	MYC
Amplification Peak 6	11q13.3	chr11:68826017-70332830	2.00E-03	2.00E-03	12	2	CCND1
Amplification Peak 7	12q15	chr12:65956377-71305401	1.01E-03	1.01E-03	37	0	MDM2
Amplification Peak 8	14q13.3	chr14:34539603-39441768	3.89E-03	3.89E-03	35	1	NKX2-1
Amplification Peak 9	19q13.2	chr19:33045997-41936165	1.55E-01	1.55E-01	243	3	
Amplification Peak 10	20q13.31	chr20:55285104-63025520	1.84E-03	1.84E-03	101	14	
Deletion Peak 1	1p36.12	chr1:1-243527304	9.14E-02	2.06E-01	2302	105	
Deletion Peak 2	1p13.3	chr1:60530721-146016270	2.67E-02	7.45E-02	429	14	
Deletion Peak 3	2q36.2	chr2:205814436-243199373	2.02E-02	2.02E-02	310	23	
Deletion Peak 4	3p21.31	chr3:1-93518806	2.20E-02	2.20E-02	608	34	FOXP1/ROBO1
Deletion Peak 5	4q35.1	chr4:159596781-191154276	2.38E-03	2.38E-03	130	6	FAT1
Deletion Peak 6	5q12.3	chr5:44966169-180915260	1.36E-01	1.33E-01	855	50	
Deletion Peak 7	6p21.32	chr6:1-171115067	4.85E-02	4.85E-02	1209	52	HLA region

Deletion Peak 8	8p22	chr8:1-35271733	1.93E-05	1.93E-05	248	16	
Deletion Peak 9	9p21.3	chr9:21692249-22534262	1.47E-15	1.47E-15	6	0	CDKN2A
Deletion Peak 10	9p13.1	chr9:35003901-69735668	9.14E-02	9.14E-02	112	6	
Deletion Peak 11	10q24.31	chr10:77154012-135534747	4.45E-02	4.45E-02	444	26	PTEN
Deletion Peak 12	12q24.31	chr12:76150197-133851895	3.69E-02	3.69E-02	443	26	
Deletion Peak 13	13q12.11	chr13:1-51828668	1.52E-04	1.52E-04	271	8	
Deletion Peak 14	15q13.3	chr15:1-46162478	1.17E-01	1.20E-01	270	11	
Deletion Peak 15	16q23.3	chr16:70201428-90354753	1.55E-02	1.55E-02	201	7	
Deletion Peak 16	17p11.2	chr17:1-25650608	5.94E-02	5.94E-02	396	18	USP22
Deletion Peak 17	18q21.32	chr18:35234114-78077248	3.99E-02	3.99E-02	172	10	SMAD4
Deletion Peak 18	19p13.3	chr19:1-3940726	1.93E-05	2.07E-04	132	5	
Deletion Peak 19	19p13.2	chr19:1-32288846	3.62E-03	2.06E-01	662	28	
Deletion Peak 20	19q13.42	chr19:11678462-59128983	1.90E-01	1.90E-01	1153	75	

Supplementary Table 6. Partial correlation between TIL abundance and MS3 proportion adjusting for histology of NSCLC.

TIL type	Spearman rank correaltion			Spearman partial correlation*	
	r	*P*	FDR q	r	*P*
B cell	0.34	0.00	0.01	0.21	0.05
CD4+ T cell	0.30	0.00	0.01	0.16	0.14
CD8+ T cell	-0.12	0.27	0.32	-0.16	0.12
Neutrophil	0.03	0.81	0.81	-0.18	0.09
Macrophage	0.23	0.03	0.05	0.07	0.54
Dendritic cell	0.22	0.04	0.05	0.04	0.70

*: adjusting for histology of NSCLC

Supplementary Table 7. Clinical information and TIL abundance of 90 NSCLC patients.

SampleID	Age	Gender	Smoking history	Histology	Chemoradiotherapy	Targeted therapy	B cell	T cell (CD4)	T cell (CD8)	N*	M*	DC*
NJLCC_0001	71	Male	Ever−Smoker	Lung SCC	chemotherapy	No	0.00	0.18	0.11	0.09	0.05	0.49
NJLCC_0003	70	Male	Ever−Smoker	Lung SCC	None	No	0.03	0.24	0.00	0.10	0.02	0.37
NJLCC_0004	71	Male	Ever−Smoker	Lung SCC	None	No	0.11	0.09	0.21	0.15	0.00	0.35
NJLCC_0005	71	Male	Ever−Smoker	Lung SCC	chemotherapy	No	0.10	0.07	0.34	0.15	0.09	0.51
NJLCC_0008	58	Male	Ever−Smoker	Lung AD	chemotherapy	No	0.00	0.17	0.19	0.13	0.25	0.85
NJLCC_0009	59	Male	Never−Smoker	Lung AD	chemotherapy	No	0.05	0.24	0.07	0.10	0.08	0.47
NJLCC_0010	51	Male	Ever−Smoker	Lung AD	chemoradiotherapy	No	0.00	0.11	0.22	0.14	0.08	0.58
NJLCC_0012	67	Female	Never−Smoker	Lung AD	chemotherapy	No	0.00	0.50	0.01	0.13	0.29	0.70
NJLCC_0013	72	Female	Never−Smoker	Lung AD	chemoradiotherapy	No	0.17	0.29	0.24	0.13	0.24	0.56
NJLCC_0014	60	Male	Ever−Smoker	Lung AD	None	No	0.11	0.28	0.20	0.18	0.03	0.80
NJLCC_0015	57	Male	Ever−Smoker	Lung AD	chemotherapy	Yes	0.07	0.09	0.13	0.08	0.17	0.48
NJLCC_0016	73	Male	Never−Smoker	Lung AD	chemotherapy	No	0.07	0.36	0.03	0.07	0.10	0.57
NJLCC_0017	58	Female	Never−Smoker	Lung AD	chemoradiotherapy	No	0.03	0.02	0.50	0.21	0.10	0.73
NJLCC_0018	59	Female	Never−Smoker	Lung AD	None	Yes	0.26	0.20	0.14	0.07	0.01	0.56
NJLCC_0019	60	Female	Never−Smoker	Lung AD	None	No	0.12	0.07	0.10	0.05	0.04	0.31
NJLCC_0020	54	Male	Ever−Smoker	Lung AD	chemotherapy	Yes	0.12	0.14	0.11	0.14	0.12	0.62
NJLCC_0021	64	Male	Ever−Smoker	Lung AD	chemotherapy	No	0.20	0.22	0.06	0.05	0.03	0.37
NJLCC_0022	75	Male	Ever−Smoker	Lung AD	None	No	0.12	0.31	0.25	0.23	0.11	0.92
NJLCC_0023	65	Female	Never−Smoker	Lung AD	None	Yes	0.23	0.10	0.18	0.14	0.11	0.33
NJLCC_0024	73	Male	Ever−Smoker	Lung AD	None	No	0.19	0.26	0.22	0.12	0.00	0.64
NJLCC_0025	65	Male	Never−Smoker	Lung AD	chemoradiotherapy	No	0.14	0.15	0.09	0.20	0.11	0.74
NJLCC_0026	64	Male	Ever−Smoker	Lung AD	chemotherapy	No	0.11	0.06	0.20	0.21	0.00	0.45
NJLCC_0027	63	Male	Ever−Smoker	Lung SCC	chemotherapy	No	0.06	0.06	0.17	0.07	0.08	0.30

NJLCC_0028	57	Male	Never−Smoker	Lung AD	None	No	0.09	0.06	0.16	0.07	0.04	0.27
NJLCC_0029	77	Male	Never−Smoker	Lung AD	chemotherapy	No	0.13	0.14	0.13	0.07	0.14	0.28
NJLCC_0030	50	Male	Ever−Smoker	Lung SCC	None	No	0.04	0.08	0.09	0.04	0.00	0.28
NJLCC_0031	44	Female	Never−Smoker	Lung AD	chemoradiotherapy	No	0.20	0.45	0.30	0.20	0.00	0.96
NJLCC_0032	59	Female	Never−Smoker	Lung AD	chemotherapy	No	0.07	0.20	0.13	0.09	0.06	0.30
NJLCC_0033	59	Female	Never−Smoker	Lung SCC	chemoradiotherapy	No	0.09	0.03	0.22	0.07	0.00	0.31
NJLCC_0034	67	Male	Ever−Smoker	Lung SCC	None	No	0.10	0.07	0.35	0.07	0.03	0.40
NJLCC_0035	73	Male	Ever−Smoker	Lung SCC	None	No	0.09	0.07	0.18	0.06	0.03	0.37
NJLCC_0036	65	Male	Ever−Smoker	Lung AD	None	No	0.02	0.31	0.12	0.14	0.49	0.31
NJLCC_0037	54	Male	Ever−Smoker	Lung SCC	None	No	0.06	0.08	0.08	0.04	0.00	0.25
NJLCC_0038	66	Male	Ever−Smoker	Lung AD	chemotherapy	No	0.16	0.14	0.13	0.08	0.17	0.42
NJLCC_0039	63	Male	Ever−Smoker	Lung AD	None	No	0.10	0.05	0.34	0.14	0.14	0.56
NJLCC_0041	55	Male	Ever−Smoker	Lung AD	chemotherapy	No	0.05	0.26	0.07	0.20	0.00	0.40
NJLCC_0042	63	Female	Never−Smoker	Lung AD	None	No	0.19	0.16	0.24	0.09	0.00	0.64
NJLCC_0043	59	Male	Ever−Smoker	Lung SCC	chemotherapy	No	0.07	0.08	0.12	0.05	0.09	0.41
NJLCC_0044	52	Male	Ever−Smoker	Lung SCC	None	No	0.28	0.26	0.30	0.09	0.00	0.38
NJLCC_0045	65	Female	Never−Smoker	Lung AD	None	No	0.15	0.21	0.25	0.07	0.33	0.51
NJLCC_0047	68	Male	Never−Smoker	Lung AD	None	No	0.09	0.00	0.40	0.12	0.17	0.65
NJLCC_0048	51	Female	Never−Smoker	Lung AD	chemotherapy	No	0.12	0.04	0.28	0.10	0.06	0.52
NJLCC_0049	65	Male	Ever−Smoker	Lung AD	chemotherapy	No	0.14	0.05	0.23	0.10	0.00	0.37
NJLCC_0050	50	Male	Never−Smoker	Lung AD	chemotherapy	No	0.18	0.01	0.51	0.14	0.03	0.60
NJLCC_0051	72	Male	Ever−Smoker	Lung SCC	None	No	0.16	0.00	0.21	0.19	0.00	0.43
NJLCC_0052	74	Male	Ever−Smoker	Lung SCC	chemotherapy	No	0.15	0.13	0.17	0.04	0.00	0.38
NJLCC_0053	62	Male	Ever−Smoker	Lung SCC	None	No	0.03	0.00	0.17	0.06	0.00	0.36
NJLCC_0054	67	Male	Ever−Smoker	Lung SCC	chemoradiotherapy	No	0.07	0.03	0.19	0.09	0.00	0.41
NJLCC_0055	51	Male	Ever−Smoker	Lung SCC	chemoradiotherapy	No	0.07	0.04	0.12	0.02	0.00	0.34

NJLCC_0057	64	Male	Never−Smoker	Lung AD	None	No	0.16	0.08	0.17	0.09	0.05	0.56
NJLCC_0058	62	Male	Ever−Smoker	Lung AD	None	No	0.06	0.05	0.14	0.06	0.04	0.29
NJLCC_0059	34	Female	Never−Smoker	Lung AD	chemotherapy	No	0.18	0.04	0.25	0.06	0.05	0.38
NJLCC_0060	43	Male	Ever−Smoker	Lung AD	chemotherapy	No	0.12	0.05	0.21	0.09	0.17	0.25
NJLCC_0061	74	Female	Never−Smoker	Lung AD	None	No	0.25	0.06	0.43	0.16	0.00	0.87
NJLCC_0062	50	Female	Never−Smoker	Lung AD	chemotherapy	No	0.14	0.09	0.23	0.11	0.06	0.50
NJLCC_0063	58	Female	Never−Smoker	Lung AD	chemotherapy	Yes	0.15	0.00	0.17	0.12	0.00	0.47
NJLCC_0064	66	Female	Never−Smoker	Lung AD	None	No	0.00	0.00	0.42	0.29	0.33	0.75
NJLCC_0065	34	Female	Never−Smoker	Lung AD	chemotherapy	No	0.17	0.21	0.17	0.12	0.02	0.54
NJLCC_0066	50	Male	Never−Smoker	Lung AD	None	No	0.14	0.09	0.21	0.10	0.06	0.53
NJLCC_0067	45	Male	Never−Smoker	Lung AD	None	No	0.16	0.11	0.10	0.12	0.11	0.50
NJLCC_0068	55	Female	Never−Smoker	Lung AD	None	No	0.09	0.06	0.28	0.13	0.09	0.55
NJLCC_0069	62	Male	Ever−Smoker	Lung AD	None	No	0.11	0.11	0.08	0.08	0.22	0.46
NJLCC_0070	57	Female	Never−Smoker	Lung AD	chemotherapy	No	0.10	0.09	0.14	0.10	0.00	0.49
NJLCC_0072	58	Male	Ever−Smoker	Lung AD	None	No	0.21	0.13	0.37	0.11	0.01	0.66
NJLCC_0073	63	Male	Ever−Smoker	Lung SCC	chemotherapy	No	0.08	0.00	0.14	0.05	0.00	0.32
NJLCC_0074	54	Male	Ever−Smoker	Lung SCC	None	No	0.08	0.06	0.34	0.07	0.00	0.36
NJLCC_0075	63	Male	Never−Smoker	Lung SCC	None	No	0.12	0.02	0.15	0.04	0.00	0.34
NJLCC_0076	65	Male	Ever−Smoker	Lung SCC	None	No	0.12	0.00	0.27	0.10	0.00	0.41
NJLCC_0077	59	Male	Ever−Smoker	Lung SCC	chemotherapy	No	0.00	0.05	0.10	0.03	0.00	0.67
NJLCC_0078	39	Male	Ever−Smoker	Lung SCC	None	No	0.04	0.01	0.11	0.03	0.00	0.32
NJLCC_0079	69	Male	Ever−Smoker	Lung SCC	None	No	0.08	0.05	0.34	0.17	0.03	0.49
NJLCC_0080	68	Male	Ever−Smoker	Lung SCC	chemotherapy	No	0.09	0.02	0.22	0.05	0.00	0.33
NJLCC_0081	64	Female	Never−Smoker	Lung SCC	chemotherapy	No	0.08	0.04	0.15	0.14	0.00	0.57
NJLCC_0082	50	Male	Never−Smoker	Lung SCC	chemotherapy	No	0.10	0.06	0.09	0.06	0.00	0.28
NJLCC_0083	66	Male	Ever−Smoker	Lung SCC	NA	No	0.07	0.03	0.12	0.05	0.00	0.27

NJLCC_0084	58	Male	Ever−Smoker	Lung SCC	chemotherapy	No	0.00	0.00	0.33	0.07	0.07	0.41
NJLCC_0085	61	Male	Ever−Smoker	Lung SCC	None	No	0.06	0.00	0.35	0.15	0.04	0.39
NJLCC_0086	73	Male	Never−Smoker	Lung SCC	NA	No	0.19	0.04	0.25	0.06	0.07	0.35
NJLCC_0087	59	Male	Ever−Smoker	Lung SCC	chemotherapy	No	0.10	0.04	0.07	0.03	0.00	0.24
NJLCC_0088	64	Male	Never−Smoker	Lung SCC	chemotherapy	No	0.09	0.04	0.11	0.02	0.00	0.30
NJLCC_0089	51	Male	Ever−Smoker	Lung SCC	chemoradiotherapy	No	0.08	0.00	0.33	0.04	0.00	0.41
NJLCC_0090	75	Female	Ever−Smoker	Lung SCC	None	No	0.05	0.00	0.40	0.17	0.17	0.70
NJLCC_0091	69	Female	Never−Smoker	Lung AD	chemotherapy	No	0.22	0.16	0.26	0.08	0.01	0.38
NJLCC_0092	50	Female	Never−Smoker	Lung AD	chemotherapy	No	0.26	0.33	0.39	0.09	0.00	0.69
NJLCC_0093	54	Male	Never−Smoker	Lung AD	chemotherapy	No	0.20	0.00	0.44	0.07	0.00	0.46
NJLCC_0094	61	Male	Ever−Smoker	Lung AD	chemotherapy	No	0.17	0.02	0.19	0.07	0.00	0.36
NJLCC_0095	68	Female	Never−Smoker	Lung AD	NA	No	0.03	0.16	0.26	0.10	0.08	0.86
NJLCC_0096	66	Male	Ever−Smoker	Lung AD	None	No	0.17	0.00	0.78	0.23	0.21	0.92
NJLCC_0097	45	Female	Never−Smoker	Lung AD	chemotherapy	No	0.19	0.05	0.17	0.04	0.00	0.32
NJLCC_0098	62	Male	Ever−Smoker	Lung AD	None	No	0.15	0.02	0.16	0.12	0.00	0.44

*: N, Neutrophil; M, Macrophage; DC, Dendritic cell

Supplementary Table 8. The primer for the detection of rearrangements in adjacent normal tissues.

Sample	Fused gene	Primer 1	Primer 2
NJLCC_0067	EML4-ALK	Left: TTCTTAGCAGCAACAGGTGG	Left: GCCAGTCCTCAATAATTCACCA
		Right: CTCCTGCCCTGTTTCCCTAA	Right: GGACTGCAGTTTCCCTCTCT
NJLCC_0031	KIF5B-RET	Left: ACATTTCAAAATTGGCTCCTGAA	Left: AGTCTTCAGTGCCTTAGTTTGC
		Right: CTTCCCTGCCGCTGTCAC	Right: ACAGTCAAGGTCAGTGTCGA
NJLCC_0066	EZR-ROS1	Left: CGCGAGAAGGAGGAGTTGAT	Left: CGTGGAGAGAGAGAAAGAGCA
		Right: CCAAAGGTCAGTGGGATTGT	Right: CAAAGGTCAGTGGGATTGTAACA
NJLCC_0059	CD74-ROS1	Left: AGGCTGGTCTTGAACTCCTG	Left: CCACCACGCCCAGCTAAT
		Right: TTAGCATGCCAAGACCAACG	Right: TGATGCATGTGAGTCCTTTAACT
NJLCC_0048	CD63-BCAR4	Left: ATCATGTTGGTGGAGGTGGC	Left: GGGCCCCTGTAATGCATAGA
		Right: GGTCTCGAACTCCTCACCTC	Right: ACAGACATAAGCACCACCTTG
